# Priming the self as an agent influences causal, spatial, and temporal events: implications for animacy, cultural differences, and clinical settings

**DOI:** 10.1007/s00426-021-01521-6

**Published:** 2021-04-28

**Authors:** John L. Dennis, Davide Margola

**Affiliations:** 1 Department of Philosophy, Social, Human & Education Sciences, Università degli Studi di Perugia, Perugia, Italy; 2 Department of Psychology, Università Cattolica del Sacro Cuore, Milan, Italy; 3 Centre for Higher Education Internationalisation, Università Cattolica del Sacro Cuore a Milano, Milan, Italy

## Abstract

People intentionally engage in goal-directed actions—i.e., set goals, create plans, and execute volitional control, which are fundamental for our understanding of ourselves, others, and events. In three experiments we created a novel sentence unscrambling task that was used to prime the self-as-agent (i.e., sentences that contain the pronoun “I”), the self-as-patient (i.e., sentences that contain the pronoun “me”), or no prime (i.e., sentences that contain proper names only), and tested whether that priming would influence the interpretation of causal, spatial, and temporal events. Results demonstrated that the self-as-agent primed participants were more likely to attribute causal influence to a kayaker in a river (Study 1), to assign spatial directionality consistent with an agent moving through space (Study 2), and to assign temporal directionality consistent with an agent moving through time (Study 3). Taken together, these three studies demonstrate that situated conceptualizations of the self as an agent can be a springboard for relevant empirical and theoretical contributions to a broad range of ideas and approaches—from theories of agency to embodied cognition, from language systems to metaphoric representation frameworks, with some potentials even in the clinical and mental health field. Along these lines, implications for animacy, cultural differences, and clinical settings are discussed.

## Introduction

People are agents not only in carrying out actions but also in exercising control over actions they perform. Perceiving oneself as having some degree of control over one’s actions has widespread importance in the development of the human species (Bruner, 1990; Metzinger & Galesse, 2003; Prinz, 1997). It plays an enormous role in our understanding and evaluation of ourselves and others (Baron-Cohen, 1995; David et al. 2008), is crucial for the formulation of the rules that govern society (Cassese, 1995), and is important for understanding mental health and well-being (Ryan et al., 2008).

We define agency as people’s ability to influence their behaviors, thoughts, and feelings to engage in goal-oriented actions (see Bandura, 2006; Baumeister et al., 2007; Leary & Tangney, 2011, for discussions of beliefs about personal control and agency). The self-as-agent can be portrayed grammatically (see Au, 1986; Ferstl et al., 2011; Fielder & Semin, 1988; Rudolph & Försterling, 1997). For example, if “*I* telephone my friend Charles,” I am not only the grammatical subject but also the agent (or cause) of telephoning; whereas, if “Charles telephones *me,*” I am the object of the telephoning act initiated by Charles. Interestingly, even when “Charles telephones *me*,” I am not completely inert or passive.

If agency is of paramount importance for our understanding of ourselves, others, and the world at large (MacMurray, 1957), then people should possess a readiness to detect agents, and indeed they do (Baron-Cohen, 1995; Heider & Simmel, 1944). The readiness to detect agents is closely related to the attribution process, in that agency attributions are typically assigned to the entity that one is attending to (e.g., Lassiter et al., 2002; Taylor & Fiske, 1978). Primed agency information has been shown to significantly influence attributions, often despite contradictory contextual information (Jones, de-Wit, Ferneyhough, & Meinz, 2008; Wegner & Wheatley, 1999).

Since detecting agents is crucial for our understanding and evaluation of ourselves and others, it is highly likely that there are simple situations where we attribute agency where there is none. Consider the classic Heider and Simmel’s (1944) study where participants were shown a film in which simple geometric figures (a large triangle, a small triangle, and a circle) moved across the screen. When participants were asked to ‘write down what happened,’ most participants interpreted the movement of the geometric figures as the purposeful actions of animate beings consistent with acting out a story. The importance of Heider and Simmel’s results is that these events appear to yield automatic, irresistible, and causal intentions, as well as animation impressions.

If we have a readiness to detect agents, then a conceptual model should flow from this. Situated conceptual frameworks—like that of Barsalou (e.g., )—offers just such a model. Barsalou’s situated conceptual framework bridges embodied cognition (e.g., Glenberg, 1997) and linguistic system theories like Lakoff’s (1992) metaphorical representation framework. Barsalou’s situated cognition model explains how goal-directed actions of agents are integrated with concepts and situational (contextual) elements. The model describes how when the situation elements and concepts are integrated properly, then effective goal-directed action occurs. For example, the concept of bicycle integrates information and representations of appropriate actions with bicycles, which includes information about different instances of bicycles (e.g., a mountain bike, a road bike, a child bike), different representations of properties (e.g., pedals), relations (e.g., feet on pedals), events (e.g., bicycling on a trail), and mental states (e.g., effort). This model explains how concepts support the effective action of agents within their behavioral context, with the result that salient and relevant information of the situation is processed both perceptually and conceptually, resulting in stored memories of the situated conceptualization.

Based on this model, Barsalou (2016b) also offers an account for social priming, whereby when we experience social situations, a situated self-conceptualization is constructed (Margola, Molgora, Vignoles, Costa, & Travagin, 2011), while offering an extensive source of pattern completion inferences that can be used on subsequent occasions. For example, previous research primed motivationally relevant goals with dieters by placing a poster promoting health near a vending machine, which in turn increased the sale of healthy food choices, and more so for dieters (Stöckli, Stämpfli, Messner, & Brunner, 2016). For this paper, our prime offered a situated conceptualizations that included previously performed goal-directed actions.

Many theorists in embodied cognition (Glenberg, 1997; Wilson, 2002) and linguistic system theorists (Lakoff & Johnson, 1980) argue for a directional connection influence whereby sensory/motor information that comes from our successful functioning in our environment (much like what is described in Barsalou’s (2016a, 2016b) situated conceptual framework) influences abstract thought. The common belief in these research circles is that representations about *time* are structured via our successful navigation through *space* (see Boroditsky & Ramscar, 2002; Clark, 1973; Lakoff & Johnson, 1980; Núñez et al., 2006). One example of the directional connection between sensory/motor information and successful functioning in our environment are two space–time metaphors—i.e., the ego-moving metaphor, where people are thought of as moving through time, and the time-moving metaphor, where temporal events are thought of as moving per se (see Boroditsky & Ramscar, 2002; Clark, 1973; Lakoff & Johnson, 1980; Núñez et al., 2006). Boroditsky and Ramscar (2002) asked participants to answer a temporally ambiguous question—i.e., “Next Wednesday’s meeting has been moved forward two days. Which day is the meeting now that it has been moved: Monday or Friday?” and they found that people who had just landed in an airport were more likely to answer with “Friday,” while people waiting to pick up someone at the airport were more likely to answer “Monday.” This research demonstrates that physically moving (or not moving) through space as an active agent influences our representation of time.

Recent research, however, has argued for a much broader understanding of how motivations can influence our representation of time. For example, de la Fuente et al. (2014) found that Moroccans reversed their mappings for the ego- and time-moving metaphors such that “Monday” was more likely to be selected for the ego-moving metaphor. Other research by Duffy & Fiest (2014) found that extroverts were more likely to adopt an ego-moving metaphor perspective than introverts, while Richmond et al. (2012) found that people who have a strong sense of personal agency (Vallacher & Wegner, 1989) were more likely to conceive of themselves as moving through time and adopt an ego-moving perspective. The impact of feelings/emotions on temporal perspectives has been highlighted with the research of Hauser et al. (2009) demonstrating that trait anger was associated with an ego-moving metaphor, while Li and Ji (2014) found that recalling an unpleasant event and anticipating a pleasant future were associated with an ego-moving perspective.

We are interested in connecting the vast research on agency, embodied cognition, and space/time motivation theories in a series of three straightforward experiments, where we tested whether a primed self-as-agent influenced not only the interpretation of temporal events, but also spatial and causal ones. While the spatial and temporal events we used in our Studies 2 and 3 have been used extensively in previous research (e.g., Boroditsky, 2000; Boroditsky & Ramscar, 2002; Duffy & Feist, 2014; Hauser, Carter, & Meir, 2009; Loeffler, Raab, & Cañal-Bruland, 2017; Richmond et al., 2012), Study 1 was designed for purposes of the current study for the interpretation of causal events.

For the self-as-agent priming, participants reconstructed sentences (i.e., sentence unscrambling task) with the subject pronoun “I”—e.g., “I kissed Mary under the bridge,” whereas, self-as-patient priming was done with the object pronoun “me”—e.g., “Mary kissed me under the bridge” (see the below description of the sentence unscrambling task in Study 1 materials). Methods like this have been used in the past to activate representations that are later used to interpret actions, events, and behaviors (e.g., Chatterjee & Rose, 2012; Förster, Liberman, & Friedman, 2008; Smith & Trope, 2006; Srull & Wyer, 1979; Wheeler et al., 2014). For example, in the seminal paper by Srull and Wyer (1979) participants completed a sentence unscrambling task that was designed to activate “hostility.” Their priming task varied the percentage of items (either 20 or 80%) that were associated with hostility, and then participants were asked to evaluate a vignette of “Donald” who exhibited a series of behaviors that were ambiguous with respect to hostility. Participants who completed the sentence unscrambling priming with 80% of the items associated with hostility were much more likely to disambiguate Donald’s behavior as hostile.

Before we turn our attention to our Studies 1–3, we will first discuss our pilot study where we tested whether our prime was effective in activating the self-as-agent.

## Pilot study

A pilot study was designed to test whether our prime activated the self-as-agent using Vallacher and Wegner’s (1989) Behavioral Identification Form (BIF). With the BIF, people can describe behaviors like “eating” in terms of the end goal of behavior (i.e., “getting nutrition”), or in terms of the sequence of individual actions that structure that behavior (e.g., “chewing” or “swallowing”). We predicted that priming the self-as-agent would engage people to describe eating as “getting nutrition” more so than “chewing” or “swallowing.” Vallacher and Wegner (1989) found that responses for Rotter’s (1966) Locus of Control (LOC), where people can agree with statements like “People’s misfortunes result from the mistakes they make,” highly correlated with BIF behavioral descriptions that emphasize the end goal of behavior—e.g., eating being described as “getting nutrition” more so than “chewing” or “swallowing.” We, therefore, designed a study where participants first completed Rotter’s (1966) LOC and then Vallacher and Wegner’s (1989) BIF so that we could determine whether differences found with the BIF were due to the prime or individual differences.

Ninety native English-speaking participants first completed Rotter’s (1966) LOC questionnaire followed by the sentence unscrambling task (for a complete description of this task, see Study 1 method) and, finally, participants completed Vallacher and Wegner’s (1989) BIF.

Rotter’s (1966) LOC consists of 29-item dichotomous response pairs of expressed belief statements that measures how much people believe they can control events. It distinguishes between individuals with a high internal locus of control who believe that events result primarily from their behavior/actions or individuals with a high external locus of control who believe that events result primarily from powerful others, fate, or chance.

Vallacher and Wegner’s (1989) BIF consists of 25-item dichotomous response pairs that assesses individual differences in action identification. It distinguishes between individuals with a low-level construal that emphasizes how to do an action and the details of the action, while individuals with a high-level construal that emphasizes why the action was performed and the action’s meaning.

The sentence unscrambling task had three conditions, i.e., sentences that included the subject pronoun “I”—i.e., the self-as-agent prime, the object pronoun “me”—i.e., the self-as-patient prime, or the control condition that included proper names only (see the below description of the sentence unscrambling task in Study 1 materials).

A one-way ANOVA was conducted to test if the scores on the LOC differed by condition. Results were not significant (*F*(2, 87) = 0.494, *p* > 0.05, MSE = 15.57), and revealed that the participants did not differ in terms of the LOC before the priming task. A one-way ANOVA was then conducted to test the effect of the self-as-agent priming on the BIF. In this case, results were significant (*F*(2, 87) = 3.738, *p* < 0.05, MSE = 15.32). Planned comparisons (Fisher’s LSD) revealed that participants identified their actions in terms of the end goal actions significantly more so following the self-as-agent prime (*M* = 17.35, SD = 2.7) than following the self-as-patient prime (*M* = 15.21, SD = 3.94, *t*(87) = 2.109, *p* < 0.05), or non-primed control group (*M* = 14.60, SD = 4.69, *t*(87) = 2.631, *p* < 0.01). The difference between control and passive self-concept was non-significant (*t* < 1), and only for those participants who unscrambled sentences that included proper names (i.e., the control condition), the scores for Rotter’s (1966) LOC and Vallacher and Wegner’s (1989) BIF significantly correlated (*r*(88) = 0.407, *p* = 0.017).

These results helped us conclude that our self-as-agent prime resulted in a higher level of action identification as indicated by Vallacher and Wegner’s (1989) BIF, and because there were no differences between conditions on Rotter’s (1966) LOC and the correlation between the BIF and the LOC was significant only for the control condition, we could safely conclude that our prime effectively activated the self-as-agent prime.

## The current studies and predictions

**Fig. 1:**
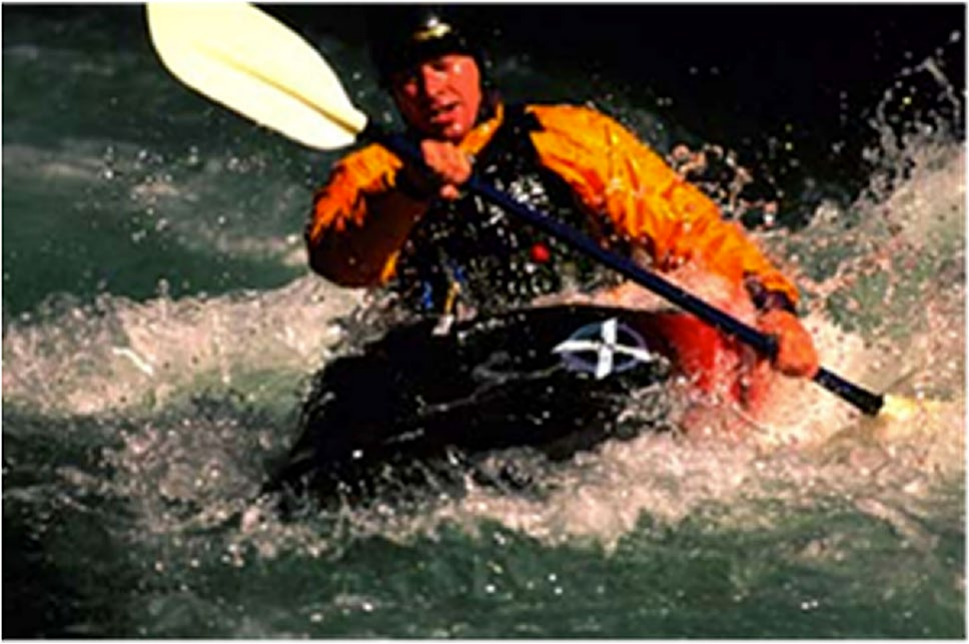
Kayaking photo example

**Fig. 2:**
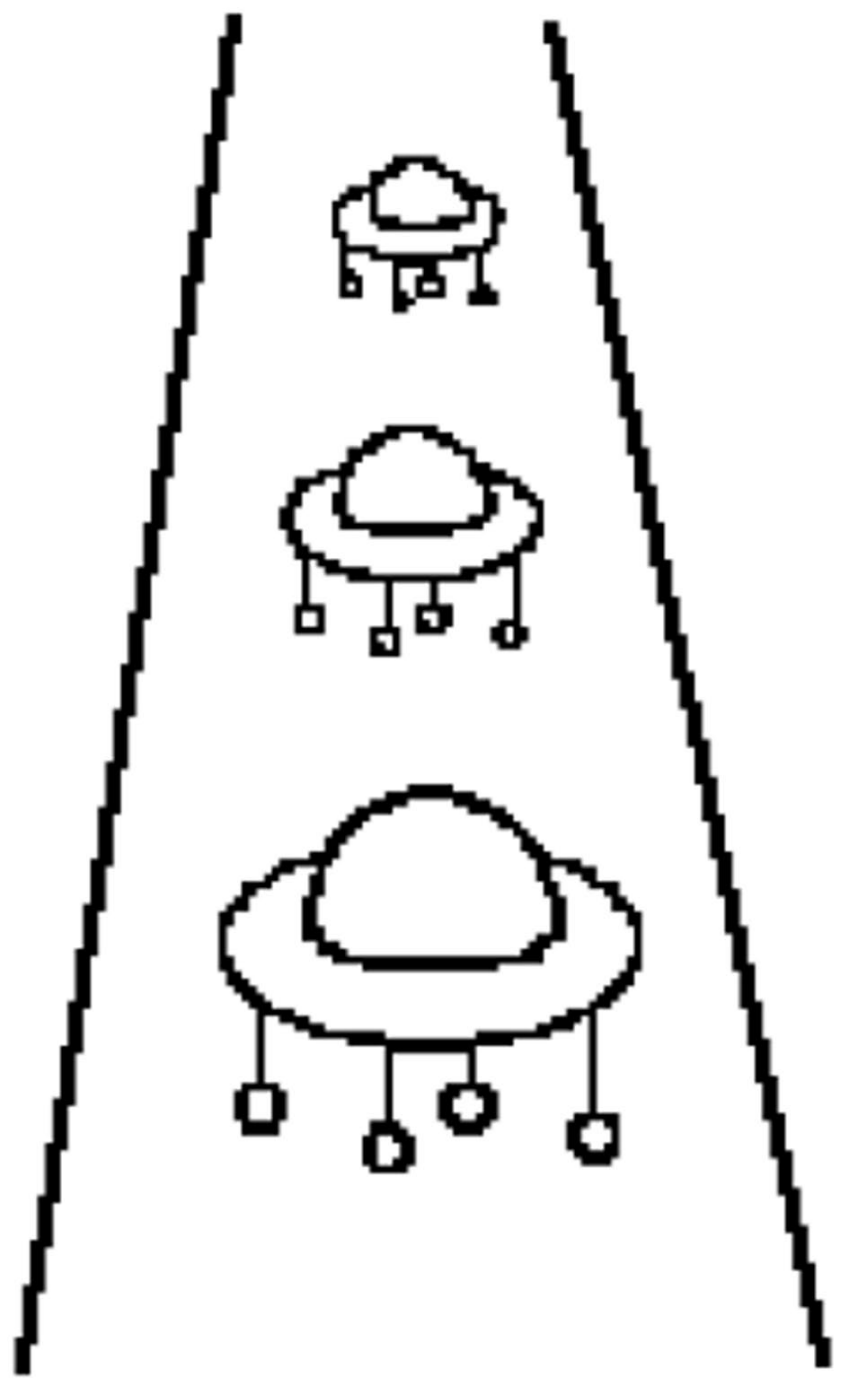
From Boroditsky (2000, p. 13)

In each of our studies, participants first completed one of three primes—i.e., self-as-agent, self-as-patient, and the control condition, then evaluated causal, spatial, and temporal events. Consistent with previous classic research that indicates that we seek intentional causes for behaviors (e.g., Heider, 1958; Jones & Davis, 1965) and Barsalou’s situated conceptual framework () that indicates how goal-directed actions of agents are integrated with concepts and situational (contextual) elements, we hypothesized that our priming of the self-as-agent would activate a “situated conceptualization” that would be used to interpret these events consistent with the prime. In Study 1, following the prime, participants evaluated a set of kayaker photos by indicating if the kayak was moved by the river’s current or by the kayaker (see Fig. 1). We predicted that participants primed with the self-as-agent would be more likely to highlight the role of the kayaker than participants in the other experimental groups. In Study 2, participants were presented with a drawing of three stylized widgets (see Fig. 2) and asked to indicate which widget was ahead. Since the widget’s shape did not indicate orientation, it was not clear if they were moving “away from” or “toward” the viewer. Contrary to what Loeffler, Raab, & Cañal-Bruland (2017), but consistent with Boroditsky (2000), we predicted that the self-as-agent primed participants were more likely than participants in the other experimental groups to take on the perspective of an agent moving through space resulting in “ahead” (i.e., away from), being assigned to the farthest away position. In Study 3, participants were presented a description of a meeting on Wednesday that had been moved forward two days and were asked to indicate which day the meeting had been moved to. Since the word “forward” is ambiguous in terms of temporal ordering (see Fig. 4), participants could have indicated either Monday or Friday. Analogous with previous embodied cognition research that demonstrated that people traveling on a plane or train were more likely to say that the meeting was moved to Friday (Boroditsky & Ramscar, 2002), we predicted that self-as-agent primed participants would be more likely than participants in the other two experimental groups to select Friday.

## Study 1

We presented participants with a causal event—i.e., a kayaker in a river. The navigation of the kayak could be primarily guided by the person or the river’s current. We predicted that the self-as-agent primed participants would be more likely to describe the kayak as being controlled by the person than participants in other experimental groups.

## Method

### Participants

Ninety-nine native English-speaking university students were randomly assigned to the primed self-as-agent, primed self-as-patient, or non-primed control group. Participants received course credit for their efforts. Sample size and statistical power of mean comparison were calculated with an alpha = 0.05, desired power = 0.80, and anticipated effect size (Cohen’s *f* = 0.34), revealed a required group size of at least 29 participants per condition, making 33/condition for each study more than adequate (Unless otherwise indicated, the analysis included herein was performed in XLSTAT; Addinsoft, 2020). (It should be noted that Study 2 was stopped at 30/condition, while Study 3 continued to run in the laboratory for one extra day (42/condition) after the pre-determined sample size was reached. In all cases, the inclusion of extra participants per condition did not alter the reported analysis.)

### Materials

A 20-page pre-test questionnaire was constructed in the English language where each page included a separate causal control evaluation task.

For the pre-test causal evaluation task, twenty photos of a kayaker in a river (e.g., Fig. 1) were pre-tested to 50 participants to ensure that they were sufficiently ambiguous in terms of control. Each photo was labeled (e.g., “Kayak Photo 1”) and was presented to participants in the same order. Participants rated the photos on a 5-point scale, where one represented “the kayak was controlled entirely by the water” and 5 represented “the kayak was controlled entirely by the person.” Only photos that had an average score between 2.5 and 3.5 were used in the subsequent experiment, resulting in seven kayaker photos being eliminated. The presentation order of the remaining 13 photos was randomized across participants.

A subsequent 14-page questionnaire was constructed in the English language. The first page contained the sentence unscrambling priming task, and the remaining 13 pages included the causal evaluation task.

For the sentence unscrambling priming task, participants re-ordered a set of words to form a sentence that made sense. This method has been used to activate representations that can later be used to interpret actions or behaviors (e.g., Chatterjee & Rose, 2012; Förster, Liberman, & Friedman, 2008; Smith & Trope, 2006; Srull & Wyer, 1979). The sentence unscrambling task consisted of 33 sentences that differed only in whether it included the subject pronoun “I” or the object pronoun “me.” Participants reconstructed sentences with the subject pronoun “I” for the self-as-agent priming, while the self-as-patient was invoked by having participants reconstruct sentences with the object pronoun “me.”

To ensure the effectiveness of the prime, interpersonal action verbs (Au, 1986; Brown & Fish, 1983) were used for the sentence unscrambling task. Interpersonal action verbs are transitive verbs where the syntactic subject is recognized as the prevalent cause of action (e.g., in the sentence ‘Steve telephoned Mark,’ Steve is the syntactic subject as well as the prevalent cause of the action of telephoning Mark). Since actions can be experienced as either positive or negative, positive, and negative valence verbs were equally used for constructing the phrases in the sentence unscrambling task (Semin & Fielder, 1988). The non-priming sentence unscrambling task used with the control participants included the same sentences as the primes except that subject and object pronouns were replaced with proper names. The presentation order of the sentence unscrambling task was randomized across participants.

For the sentence unscrambling task, participants were presented with the following instructions: “Use the scrambled words to create a complete sentence. Write that sentence on the line below the scrambled words. Be as accurate and as fast as possible.”

### Design

The study used an independent group design with three levels: self-as-agent, self-as-patient, and no priming, while rating the kayaker photos was the dependent variable.

### Procedure

Participants were told that the experiment session involved the execution of multiple experiments to help mask the relationship between the priming task and the kayaker photo evaluation task. Participants completed the sentence unscrambling priming task and then evaluated the 13 kayaker photos. Participants in the control group completed a non-priming sentence unscrambling task and then evaluated the kayaker photos. Participants completed the experiment individually and without a time limit, but all finished within one hour. Finally, a post-experimental awareness questionnaire was administered (Bargh & Chartrand, 1999). None of the participants were excluded from the analysis because of priming or experimental design awareness.

## Results

The rating scale for the kayaker pictures was converted in the following way: the lowest point of the scale (1) “the kayak is controlled entirely by the water” was converted to a − 2, the highest point of the scale (5) “the kayak is controlled entirely by the person” was converted to a 2, and the mid-point (3) “the kayak is controlled equally by the water and by the person” was converted to a 0. An average for the 13 photos was calculated for each participant and that average was used in the below analysis.

A one-way ANOVA was conducted to test the effect of the self-as-agent vs. self-as-patient priming on the kayaking photos assessments. The result was significant (*F*(2, 96) = 5.995, *p* = 0.004, MSE = 0.077). Consistent with our prediction, self-as-agent primed participants were more likely to describe the kayak as being controlled by the person than the participants in the other experimental groups. Planned comparisons (Fisher’s weighted LSD) revealed that the self-as-agent condition (*M* = -0.119, SD = 0.246) differed significantly from the control group (*M* = − 0.308, SD = 0.287, *t*(96) = 2.772, *p* = 0.018, Cohen’s *d* = 0.707), as well as from the self-as-patient condition (*M* = − 0.336, SD = 0.295, *t*(96) = 3.183, *p* = 0.006, Cohen’s *d* = 0.799), while the difference between the self-as-patient condition and the control group did not differ (*t*(96) = 0.411, *p* = 0.911) (Table 1).

**Table 1:** Mean response for whether the kayak was controlled by the person or the water following the prime

Condition	Kayak controlled by the person
Self-as-agent	− 0.119 (0.246)
Control	− 0.308 (0.287)
Self-as-patient	− 0.336 (0.295)

Scale: 2 = kayak controlled entirely by person, − 2 = kayak controlled entirely by water, (standard deviation in parenthesis)

The estimated JZS Bayes factor (null/alternative) for the comparison between the control vs. self-as-agent primed participants was 3.46, in favor of the alternative hypothesis, while for the comparison between the self-as-patient primed participants vs. control the estimated JZS Bayes factor was 1.35, in favor of the null hypothesis, and finally, the comparison between the self-as-agent vs. self-as-patient primed participants yielded a JZS Bayes of 6.80, which was in favor of the alternative hypothesis. The first and third comparisons suggested that it was 3.46 and 6.80 times more likely to occur under the model including the effect of the prime, rather than under the model without it.

## Study 2

Typically, with a still image, directional movement is indicated by the orientation of the object, as the front of an object is often what moves forward. If instead an object is not equipped with a front (a classic example is the Zündapp Janus vehicle, 1957–8), then the directional movement is inferred contextually.

Study 2 was designed to extend the findings of Study 1 to a spatial event that was ambiguous in terms of directional movement. The event used by Boroditsky (2000) included three stylized widgets organized in perspective where the widgets’ shape did not indicate orientation thus making “aheadness” ambiguous. Indicating which widget was ahead required participants to infer directional movement from the context. We predicted that self-as-agent priming would alter the contextual information available to participants (Barsalou, 2003, 2009, 2016a). Consistent with theories of metaphorical event representations (Lakoff, 1992), as well as research on embodied theories of cognition (e.g., Glenberg, 1997), we predicted that self-as-agent primed participants would be more likely to take on the perspective of an agent moving forward through space (i.e., ahead and towards the stimulus display) compared to participants in the other experimental groups, resulting in “ahead” being assigned to the farthest away position.

## Method

### Participants

90 native English-speaking university students were randomly assigned to the primed self-as-agent, primed self-as-patient, or non-primed control group. Participants received course credit for their efforts, and none of the participants were excluded from the analysis.

### Materials

The sentence unscrambling priming task was identical to that used in Study 1.

An image of three frontless mobile-looking widgets that were arranged in perspective and therefore ambiguous in terms of “aheadness” was presented to participants. Underneath the image of the widget was the question: “Which of these widgets is ahead? (Please circle one)” (Fig. 2).

### Design and procedure

The study design and procedure for Study 2 were identical to Study 1 with one exception, i.e., the spatially ambiguous rating question was the dependent variable.

## Results

**Fig. 3:**
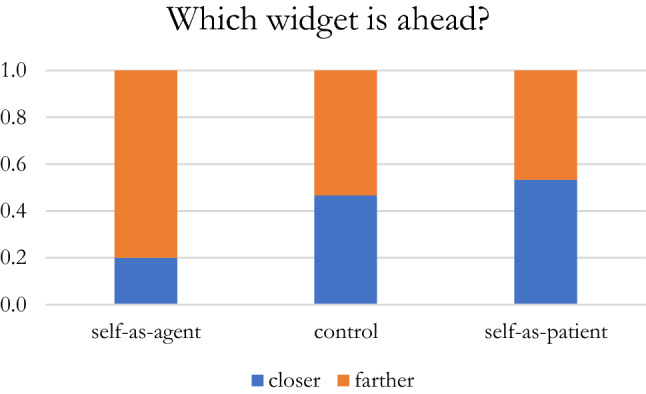
Proportion of responses for selecting which widget, i.e., the “closer” or the “farther” one, is ahead following the prime

A logistic regression tested the effect of the self-as-agent vs. self-as-patient on the “Which of these widgets is ahead?” question. 53.33% of the control group participants chose the farthest widget as “ahead,” compared to 46.67% of the self-as-patient primed participants, and 80% of the self-as-agent primed participants. When compared with a constant-only model, the results were statistically significant (*X*^2^(2) = 7.78, *p* = 0.02), indicating that the priming significantly affected participant responding. Further analysis of the regression coefficients revealed that the odds of choosing the farthest widget as “ahead” for the self-as-agent primed participants was significantly greater than 1:1 (*z* = 3.04, *p* = 0.02). The odds of assigned “ahead” to the farthest away position was greater for the self-as-agent primed participants by a multiplicative factor of 4.57 (*z* = 2.60, *p* = 0.009) when compared to the self-as-patient primed participants, by a factor of 3.50 (*z* = 2.16, *p* = 0.03) when the self-as-agent primed participants were compared to the control condition participants, and by a non-significant factor of 1.36 (*z* = 0.52, *p* = 0.29) when the self-as-patient primed participants were compared to the control condition participants (Fig. 3).

The estimated JZS Bayes factor (null/alternative) for the comparison between the control vs. self-as-agent primed participants was 2.24 in favor of the null hypothesis, while for the comparison between the self-as-patient primed participants and the control the estimated JZS Bayes factor was 1.08, again in favor of the null hypothesis, and finally, the comparison between the self-as-agent and the self-as-patient primed participants yielded a JZS Bayes factor of 2.86, which was in favor of the alternative hypothesis. This latter comparison suggested that it was 2.86 times more likely to occur under the model including the effect of the prime, rather than under the model without it.

## Study 3

**Fig. 4:**
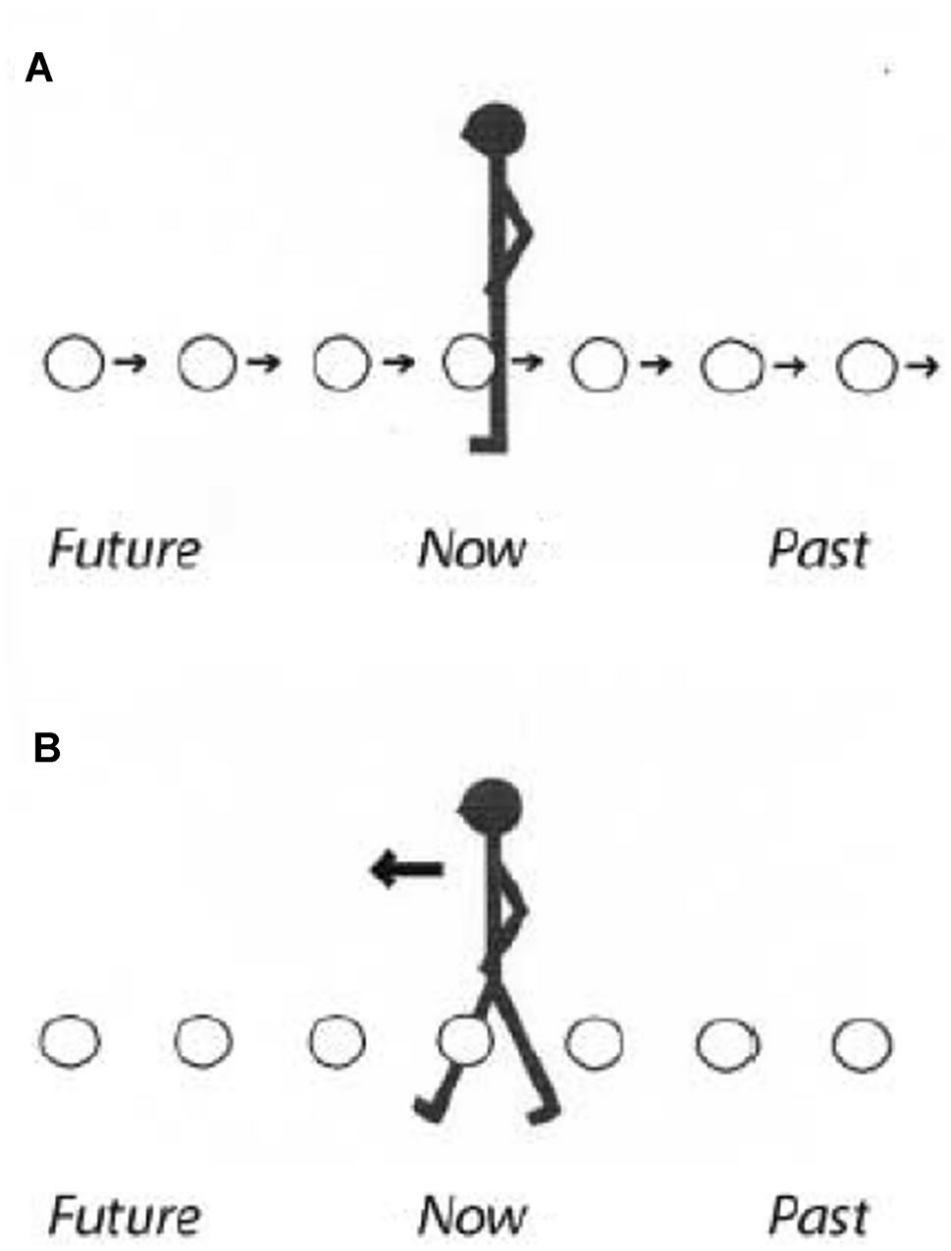
From Núñez et al. (2006, p. 134). **a** “Time-moving” metaphor. **b** “Ego-moving” metaphor

Temporal events can be ordered in many ways. One is in relation to a person(s), e.g., “The meeting is fast approaching,” “We are fast approaching the meeting.” In the English language, the former is an example of the time-moving metaphor and the latter of the ego-moving metaphor (Clark, 1973; Lakoff & Johnson, 1980) (Fig. 4). With the ego-moving metaphor (Fig. 4b) people are thought of as moving through time, whereas with the time-moving metaphor (Fig. 4a) temporal events are thought of as moving per se.

Study 3 extended the findings from Studies 1 and 2 to a temporal event (Boroditsky & Ramscar, 2002; Núñez et al., 2006). The temporally ambiguous event “Next Wednesday’s meeting has been moved forward two days. Which day is the meeting now that it has been moved: Monday or Friday?” is ambiguous in that “forward” could be intended to mean forward to either Monday or Friday.

Boroditsky and Ramscar (2002) found that relatively mobile people, e.g., just landed in an airport, were more likely to answer the temporally ambiguous question with “Friday,” while relatively immobile people, e.g., waiting to pick up someone at the airport, were more likely to say “Monday.” Extending the above research by Boroditsy & Ramscar (2002) and consistent with the research of Richmond et al. (2012) who found that people who have a high level of personal agency (Vallacher & Wegner, 1989) were more likely to conceive of themselves as moving through time and adopt an ego-moving perspective, we predicted that the self-as-agent primed participants would be more likely to have an ego-moving understanding of time (Fig. 4b) than participants in the other experimental groups.

## Method

### Participants

126 native English-speaking university students were randomly assigned to the primed self-as-agent, primed self-as-patient, or non-primed control group. Participants received course credit for their efforts, and none of the participants were excluded from the analysis.

### Materials

The sentence unscrambling priming task was identical to that used in Study 1.

A statement that described the temporal movement of an event: “Next Wednesday’s meeting has been moved forward two days” was presented to participants. Below the event description was the question: “Which day is the meeting now that it has been moved: Monday or Friday? (Please circle one).”

### Design and procedure

The design for Study 3 was identical to the previous studies with one exception, i.e., the temporally ambiguous rating question was the dependent variable.

## Results

**Fig. 5:**
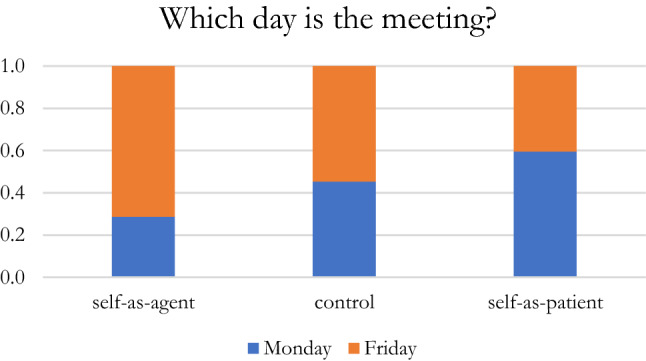
Proportion of responses for selecting which day is next Wednesday’s meeting now that it has been moved forward 2 days following the prime

A logistic regression was used to test the effect of priming on the question “Next Wednesday’s meeting has been moved forward two days. Which day is the meeting now that it has been moved: Monday or Friday?” 54.48% of the control group participants chose “Friday”, compared to 40.05% of the self-as-patient primed participants, and 71.4% of the self-as-agent primed participants, with the latter being consistent with the ego-moving metaphor (Boroditsky & Ramscar, 2002; Clark, 1973; Lakoff & Johnson, 1980; Núñez et al., 2006). When compared with a constant-only model, the results were statistically significant (*X*^*2*^(2) = 8.16, *p* = 0.02), indicating that the priming significantly affected participant responding. Further analysis of the regression coefficients revealed that the odds of choosing “Friday” as the day for the new meeting for the self-as-agent primed participants was significantly greater than 1:1 (*z* = 2.68, *p* = 0.007). The odds of assigning “Friday” for the self-as-agent primed participants was greater than the self-as-patient primed participants by a multiplying factor of 3.68 (*z* = 2.81, *p* = 0.005), by a non-significant factor of 2.07 (*z* = 1.57, *p* = 0.12) when the self-as-agent primed participants were compared to the control condition participants, and by a factor of 1.78 (*z* = 1.32, *p* = 0.08) when the self-as-patient primed participants were compared to the control condition participants (Fig. 5).

The estimated JZS Bayes factor (null/alternative) for the comparison between the control vs. self-as-agent primed participants was 1.62 in favor of the null hypothesis, while the estimated JZS Bayes factor for the comparison between the self-as-patient primed participants and the control was 1.48, again in favor of the null hypothesis, and finally, the comparison between the self-as-agent and the self-as-patient primed participants yielded a JZS Bayes factor of 2.78, which was in favor of the alternative hypothesis. This latter comparison suggested that it was 2.78 times more likely to occur under the model including the effect of the prime, rather than under the model without it.

## General discussion and conclusions

This article reports evidence indicating that self-as-agent priming can be transferred to causal, spatial, and temporal events. We used Barsalou’s (2003, 2009, 2016a, 2016b) situated conceptual framework to formulate our hypotheses, whereby information from the self-as-agent prime would activate correspondingly relevant situated conceptualizations that can guide the processing of different situations. In this direction, self-as-agent primed participants would be more likely to offer agentic interpretations to causal, spatial, and temporal events when compared to self-as-patient and non-primed control participants.

Study 1 asked participants to describe a kayak as being controlled by the water or by the person. Similar to research on the inferential character of self-concept (e.g., Aaker & Lee, 2001; Bargh et al., 1988; Margola et al., 2011), causal interpretations of the kayaking photos were inferred from the primed self-as-agent information. Consistent with Barsalou’s (2016a) conceptual framework, Lakoff’s (1992) metaphoric representation theory, and Glenberg’s (1997) embodied cognition model, self-as-agent priming was more often associated with a description of the kayaker as being moved by the person than the other two conditions. In other words, self-as-agent primed participants gave the kayaker a more active role in navigating the river, while the non-primed and self-as-patient primed participants were more likely to see the kayak as moved by the water.

Study 2 asked participants to disambiguate “aheadness” of a set of three widgets as they could have been moving “away from” or “toward” the viewer. Extending and consistent with situated representation theories (Barsalou, 2003, 2009) as well as metaphoric representation theories (Lakoff, 1992), self-as-agent priming was more likely than passive and non-primed participants to assign “ahead” to the widget in the farthest away position, that is, away from the viewer. This position is the only one that is consistent with the representation of a goal-oriented agent moving through space (e.g., Lakoff, 1992).

For Study 3, participants were asked to evaluate the ambiguous “forward” movement of a temporal event from Wednesday to either Monday or Friday. Extending embodied cognition theories and research on temporal metaphors (e.g., Boroditsky & Ramscar, 2002; Núñez, Motz, & Tuescher, 2006), self-as-agent priming assigned a “forward” movement from Wednesday to Friday consistent with an ego-moving metaphor (persons dynamically moving through time and showing a high level of personal agency).

The fact that the self-as-patient primed participants did not assign movement “consistent” with their prime—i.e., view the kayaker as being moved by the water (Study 1), view the closest widget as being the one that is ahead (Study 2), and move Wednesday’s meeting forward to Monday (Study 3), is compelling. In other words, our failure to find a consistent difference between the self-as-patient primed participants and the control group could be because their prime did not do a good job of activating a representation of the self as “inert” or because the self-as patient is the default, therefore leading to a ceiling effect.

Altogether, Studies 1–3 demonstrated that the perspective taken by participants for the causal (kayaker), spatial (widget), and temporal (meeting) events were primarily altered by the self-as-agent prime. It is entirely possible that if participants evaluated different situations, self-as-agent priming would yield different results. For example, in research we have conducted we find that if the causal event is *negative* like hitting someone with one’s car, the priming of a self-as-agent was associated with people taking the perspective of being *less* in control, not *more* (Dennis, Margola, Provost, & Capurso, manuscript in preparation). Similarly, if the spatial event or temporal event is *positive* like moving towards home or an appointment to see a friend, priming of self-as-agent would be associated with people taking the perspective of moving those spatial and temporal events *towards* as opposed to *away* from the self (Dennis, Margola, Provost, & Capurso, 2020 manuscript in preparation). Since the representation of the self-as-agent is entangled with our understanding and representation of goal-oriented action, any movement, be it biological or not, could be influenced by the prime that we have designed—consistent with Barsalou’s (2003, 2009, 2016a, 2016b) situated cognition framework.

That said, it is important to understand how the representation of the self-as-agent would influence events. As discussed previously, in Heider and Simmel’s (1944) study, 120 participants were shown a film where simple geometric figures (large triangle, small triangle, and circle) moved across the screen. When participants were asked to ‘write down what happened,’ only three of them said something like “I see geometric figures moving across the screen,” while 117 participants saw a story, where the movement of the geometric figures is embodied with purposeful actions of animate beings. While this is a wonderful example of our nearly effortless storytelling process (TRBQ, 2014), such that it seems that we were made to tell stories (Gottschall, 2012), it is also an example of our nearly effortless imbuing of agency when telling those stories of events. Therefore, would the self-as-agent primed participants be more likely to describe the goals of the agents (e.g., trying to catch) vs. self-as-patient participants (e.g., running away from) in these films? Or consider Michotte’s (1963) causal event where one ball “causes” the movement of another ball. Would the self-as-agent primed participants be more likely to describe the event in terms of agents (A ball hit B ball) vs. self-as-patient participants (B ball was hit by A ball)? Since Michotte’s causal events yield automatic, irresistible, causal, and animacy impressions—if the self-as-agent primed participants would describe these events differently than non-primed participants, it would demonstrate how much higher-level cognitive processing can influence seemingly perceptual phenomenon.

Several questions remain. These include the sort of events that can be influenced by this prime, the valence (positive/negative) of the behaviors being evaluated, the default interpretation of those events/behaviors, and the temporal stability of the prime (how long does it last?). Given the importance of agency representations in our understanding and evaluation of ourselves and others, we see the use of self-as-agent priming as a useful tool to explore these issues. For example, it would be important to know if this prime would interact with culture for the interpretation of numerous events—e.g., internally vs. externally driven events. In this perspective, Morris et al. (1995) presented five fish in a scene such that one fish is ahead and four are grouped behind. Chinese participants were more likely than USA participants to interpret the event as being driven by external forces, while USA participants were more likely than Chinese participants to interpret the event as being driven by internal forces. Self-as-agent priming could very well interact with one’s culture by driving more internal force interpretations of this event.

Finally, our results echo the foundations of most schools and orientations in clinical practice, where the key purpose of therapy is to help clients take responsibility and to move beyond fate or chance (see Study 1), as well as switching perspective based on insightful and contextual information (see Studies 2 and 3). Moving from a self-as-patient perspective to a self-as-agent, with it’s associated higher level of action identification (see Pilot Study) is beneficial in clinical work (Clarkin & Levy, 2004), where there is a movement from the self-as-patient (here “patient” meant literally) to a self-as-agent view while elaborating “our formal and informal theories of the world” (Slife, 2004, p. 76). Consistent with this perspective, in another line of research (Margola et al., 2018), we found that self-distancing from negative past events reduced stress, and much like the research of Li and Ji (2014), we think that taking on the view of the self-as-agent or the ego-moving perspective might be just what is needed to increase causal, spatial, and temporal self-distancing, as well as personal well-being and psychological health.

In sum, the studies presented in this paper offer insight into how a self-as-agent prime influenced the evaluation of causal, spatial, and temporal events. Indeed, we seek intentional causes for behaviors, and our prime assisted people in doing just that. The studies in this paper help unite research on agency/animacy (e.g., Heider, 1958; Jones & Davis, 1965) with Barsalou’s situated conceptual framework (; b), as well as research on embodied cognition (Glenberg, 1997), linguistic system theories (Lakoff & Johnson, 1980), and metaphorical representations of space/time (Clark, 1973), while offering a novel priming that can be used in future research.
